# An impact of HIV disease severity on in vitro CD4+ T cell expansion using anti-CD3/28 coated magnetic beads for being used as an alternative treatment in HIV infected patients

**DOI:** 10.1186/1471-2334-14-S2-P66

**Published:** 2014-05-23

**Authors:** Nattawat Onlamoon, Vajee Petphong, Kasama Sukapirom, Kovit Pattanapanyasat

**Affiliations:** 1 Mahidol University, Bangkok, Thailand

## Introduction

Antiretroviral therapy (ART) could lower viral burden and increase CD4+ T cell level in HIV infected patients. However, the immune reconstitution never completes therefore an alternative treatment is required. Interestingly, an in vitro expansion of CD4+ T cells from patients through CD3/28 signaling provided intrinsic control of viral replication and transfusion of autologous expanded cells in ART treated patients showed increase in CD4 count. Since functions of CD4+ T cells were different in each stage of the disease, we determined whether disease severity has any impact on an in vitro expansion of CD4+ T cells and conducted the experiments to increase CD4 expansion level for being use in clinical trial.

## Material and methods

Whole blood samples were collected from both healthy subjects and different groups of ART treated HIV infected patients based on CD4 count (<200, 200-500 and > 500 cells/µl). CD4+ T cells were isolated using immunorosette formation and purified CD4+ T cells were expanded using anti-CD3/28 coated magnetic beads for 3 weeks. Fold expansion, viability and purity of anti-CD3/28 expanded CD4+ T cells were observed. Improvements in fold expansion of CD4+ T cells collected from patients were tested by a scheduling IL-2 supplemented culturing method.

## Results

The results demonstrated that purified CD4+ T cells from healthy subjects can be expanded up to 1000-fold after 3 weeks with high viability (>90%) and high purity of CD4+ T cells (>95%). Although highly purified expanded CD4+ T cells can be obtained from patients, a lower fold expansion was demonstrated when compare to normal subjects. Furthermore, patients with low CD4 count showed lower fold expansion when compare to patient with high CD4 count. Finally, the result showed that a low level IL-2 supplementation after 1 week of cell expansion can increased the level of cell expansion for patients.

## Conclusions

A large scale in vitro expansion of CD4+ T cells from HIV infected patients could support an immunotherapy using autologous transferred of anti-CD3/28 expanded CD4+ T cells. This method could be used as a new therapeutic strategy in HIV infected patients.

